# Teacher Teleworking during the COVID-19 Pandemic: Association between Work Hours, Work–Family Balance and Quality of Life

**DOI:** 10.3390/ijerph18147566

**Published:** 2021-07-16

**Authors:** Pablo A. Lizana, Gustavo Vega-Fernadez

**Affiliations:** Laboratory of Morphological Sciences, Instituto de Biología, Pontificia Universidad Católica de Valparaíso, Valparaíso 2373223, Chile; gustavo.vega@pucv.cl

**Keywords:** pandemic, COVID-19, schoolteachers, teleworking, mental health

## Abstract

Background: Teachers worldwide had to reinvent their work routine according to teleworking during the COVID-19 pandemic, a work format that negatively impacts individuals’ physical and mental health. This study evaluates the association between work hours, work–family balance and quality of life (QoL) among teachers during the Chilean health emergency of the COVID-19 pandemic. Teachers from across Chile were contacted via email and social media to answer an online survey. QoL was evaluated via the SF-36 questionnaire, work hours and work–family balance in the pandemic. A total of 336 teachers from across Chile participated in this study. Teachers had a low QoL score, associated with age (*p* < 0.05). Teachers who were ≤44 showed lower deterioration risks in the Physical Component Summary (OR: 0.54) than the ≥45-year-old age group; simultaneously, the younger group (≤44 years) had a greater risk (OR: 2.46) of deterioration in the Mental Component Summary than teachers over 45 years. A total of 78.7% of teachers reported having increased their work hours during the COVID-19 pandemic due to teleworking and 86% indicated negative effects on their work–family balance. Pandemic work hours and negative work–family balance increase the risk of reducing the Mental Component Summary (OR: 1.902; OR: 3.996, respectively). Teachers presented low median QoL scores, especially in the Mental Component Summary, suggesting that it would be beneficial to promote a better workload distribution for teachers in emergency contexts, considering the adverse effects of teleworking.

## 1. Introduction

In December 2019, a respiratory disease was detected in the city of Wuhan, China; on 9 January 2020, Chinese health authorities via the World Health Organization (WHO) declared it to be a novel coronavirus [1]. On 12 January 2020 the genetic sequence of what we know as COVID-19 was made public and on 11 March 2020 a global declaration was made that the infection met all the characteristics of a pandemic [2].

The first measures in affected countries were confinement, social distancing and quarantines [2,3,4,5]. In this sense, these measures obligated various labor groups to implement teleworking [6], a work format which has been associated with psychosocial risks due to increased occupational stress [7,8]. Furthermore, these work conditions generate significant problems between personal and working life [7,9] due to the lack of control over working time and family attention at home affecting work–family balance [10]. The health measures for the COVID-19 pandemic (especially quarantines) were also reported to have negative effects on the mental health of people, promoting panic, anxiety, depression, frustration, rage, boredom and paranoia [11,12,13]. This background, along with the catastrophic context generated by the health emergency, causes people to develop mass hysteria, bringing negative consequences for mental and physical health [12,14]; this manifests in diminished hours of physical activity and sleep, and increased alcohol and tobacco use [15].

Social distancing and confinement also led to school closures, generating major impacts on national dynamics [16,17,18,19]. School closures by May 2020 left 1.2 billion students worldwide without in-person classes [18]. In Chile, the suspension of classes and closure of schools, daycares and universities was declared on 16 March 2020 [20]. Along with class suspensions in Chile, from 25 March there were confinement measures, mobility restrictions and quarantines [20].

School closures generated major challenges for teachers who had to reinvent their work methodology around teleworking, immediately learning to work with necessary technology for online courses and modifying their teaching and learning model by adjusting it to the pandemic context [18,21,22]; along with exposing their personal spaces (home) and personal contact for student and parent/guardian attention in expanded timeframes [23]. This teleworking context for teachers led to various consequences in health conditions including depression, anxiety, stress and burnout syndrome due to work overload during the pandemic [24,25,26].

The impact of teleworking during the COVID-19 pandemic among Chilean teachers is reported to have contributed to development of anxiety and stress, with high workloads, exhaustion and burnout [27]. Other reports in Chile, Latin America and the Caribbean reveal that teacher teleworking during the COVID-19 pandemic has meant greater time demands for class preparation and higher stress added to family worries [18]. Furthermore, before the COVID-19 pandemic, one of the professions with the greatest deterioration in health worldwide was teaching [28,29,30], with significant increases in the deterioration of mental health and physical discomfort in professional practice, with consequent psychosocial deterioration and quality of life (QoL) disorders derived from occupational stress [31,32,33,34] and burnout [35,36,37]. In this regard, recent studies have reported a decrease in QoL during the health crisis in teachers compared to QoL scores before the pandemic [38].

Given that teachers worldwide have been considered a high-risk population for rates of professional ailments impacting their QoL since before the pandemic, there are studies on the factors that impact Chilean teachers’ physical and mental health during the COVID-19 pandemic [31,33,34,38,39,40]. Therefore, the main objective of this study was to evaluate the association between the intensity of teleworking hours during the pandemic, work–family balance and quality of life in Chilean teachers during the COVID-19 health emergency.

## 2. Materials and Methods

### 2.1. Participants

A cross-sectional study was conducted between the months of July and October 2020, a period of confinement and teleworking for teachers. A total of 362 teachers made up our initial sample, with the inclusion criteria of all teachers who had teleworking with primary or secondary school students; 14 teachers were excluded from the sample due to not satisfactorily completing all instruments, with another 12 were excluded for being university professors or teachers in higher learning institutes.

The teachers considered in the final sample were from 14 of the 16 current regions in Chile. The majority came from the Valparaíso Region (57.44%), followed by the Metropolitan Region (15.77%) and the Arica and Parinacota Region (10.42%).

### 2.2. Instruments

#### 2.2.1. Sociodemographic Information

Teachers provided information about their age, gender, marital status, region and city of residence within Chilean territory.

#### 2.2.2. Work-Related Information

Participating teachers provided information about the funding type of the establishment where they worked (public, private with state subsidies/charter school, or private without state subsidies) and work contract type (fixed-term or indefinite). Regarding the realization of telework, they also replied whether they were working 1 = more or 2 = fewer or the same number of hours as before the pandemic, considering the hours where they gave classes and all the hours in which teachers prepare materials, revise and create tests, supporting students and parents/guardians and other administrative work typical of the profession. All included teachers were teleworking.

#### 2.2.3. Personal/Family Life Information

Personal information provided by teachers was related to the impact of telework on work–family balance. In this question they had to answer 1 = if family and personal relations were affected as a result of teleworking; 2 = family and personal relations were not affected by teleworking.

#### 2.2.4. Quality of Life

The SF-36 survey, which has been adapted syntactically and semantically to the Chilean idiosyncrasy [41] and validated in Chilean teachers [38], was applied to evaluate QoL. The instrument consists of 36 questions measuring 8 health dimensions: physical function, bodily pain, role limitations related to physical problems, general health perception, vitality, social functioning, role limitations due to emotional problems and mental health, which are finally grouped into two summary measurements: Physical Component Summary and Mental Component Summary [42].

Survey score results are measured on a scale of 0 to 100, after which a t-score value is calculated with a median of 50 and a standard deviation of 10 for each of the 8 dimensions, which are categorized in the two summary measurements, Physical Component Summary and the Mental Component Summary. To calculate the t-score value, standardized values are used according to the method recommended by the creator and values greater or lesser than 50 indicate higher or lower QoL, respectively [43]. Regarding the reliability of the scale, the Cronbach’s alpha coefficient was α = 0.885 for the physical function scale, α = 0.880 for the limitations due to physical problems scale, α = 0.878 for the bodily pain scale, α = 0.874 for the general health perceptions scale, α = 0.867 for the vitality scale, α = 0.868 for the social functioning scale, α = 0.878 for the role limitations due to emotional problems scale, α = 0.865 for the mental health scale, α = 0.877 for the Physical Component Summary and α = 0.864 for the Mental Component Summary.

### 2.3. Procedure

Teachers were contacted by email and social media (Facebook and Instagram) following a snowballing approximation. Those who accepted participation proceeded to respond to the online instruments. The survey platform used was SurveyMonkey (SurveyMonkey, San Mateo, CA, USA).

Prior to data collection, each participant had to read and sign informed consent, which invited them to voluntarily participate in the study in a fully confidential way, without pay, compensation or conflict of interest with the researchers. To this regard, this study fulfills all the ethical requirements of the Helsinki Declaration, approved by the Bioethics Committee at Pontificia Universidad Católica de Valparaíso (n°BIOEPUCV-H 393-2021).

### 2.4. Statistical Analysis

The associations of sociodemographic variables were evaluated between each gender and age category and the 50th percentile of the Physical and Mental Component Summary via the chi-squared test and Fisher’s exact test. Age was categorized according to scores on the National Health Survey 2009–2010 (≤44 years old and ≥45 years old) from the Chilean Health Ministry [44]. QoL scores on their eight scales and the two summary measurements are described in the total sample, following which comparisons are made of medians between genders and age categories via *t*-tests for parametric variables and Wilcoxon tests for non-parametric variables according to the Shapiro–Wilk normality test. The 50th percentiles (50p) of each summary factor (Physical and Mental Component Summary) were used as a cutoff point to dichotomize the data. The subjects were classified in low (below 50p) or fair/good (above 50p) categories. Finally, three logistical regression models were made with the physical and mental health QoL measurements as dependent variables to evaluate the association with the following variables. First, with work hours in the pandemic and the work–family balance variable were used in the first model; the domestic work hours variable was incorporated in the second model; and, in the final one, age and gender variables were added. The goodness of fit of each logistical regression model was proven with the Hosmer-Lemeshow test. Data were analyzed with STATA 16 statistical software (2017, Stata Corp. LLC, College Station, TX, USA).

## 3. Results

### 3.1. Sociodemographic Characteristics

The total sample consisted of 336 teachers (79% women, *n* = 265) with a median age of 37.5 ± 10.7 years, with no significant differences observed between age medians between both genders. Table 1 shows the sociodemographic characteristics in the total sample after which the associations of these variables are evaluated between gender and age categories. A significant association was observed between age and marital status (*p* < 0.01) with a greater prevalence of single people in the ≤44-year-old group and age with the variable contract type, where teachers ≥ 45 years old had a higher percentage of indefinite contracts.

### 3.2. Quality of Life

In Table 2 we can observe the sociodemographic characteristics associated between the lowest and highest scores of the physical and mental health QoL components according to the 50th percentile. The group of teachers who were ≤44 years is significantly associated (*p* < 0.01) with a low score (score < p50) on the mental QoL component, with a prevalence of 84% versus 16% in the second age group (≥45 years). However, in the physical health component the first age group is significantly associated (*p* < 0.01) with a high QoL score (score > p50) and a prevalence of 82%. A significant association is also observed (*p* < 0.01) between lower scores (<p50) on the mental QoL component and the teachers reporting a high hourly workload (higher than before the pandemic) and those with their work–family balance affected.

Table 3 shows us the median values of the eight QoL scales and the two physical and mental health components according to the total sample, gender and age groups. In the total sample, the lowest value is found in the mental health (35.31 ± 10) scales, followed by the social functioning (35.6 ± 11.8) and Mental Component Summary (35.66 ± 9.5). In comparisons between both genders there are significant differences (*p* < 0.01) in four scales, physical function, bodily pain, vitality and mental health, with lower scores in the female gender. We can observe that from the summary measurements, the Mental Component Summary has the lowest score in both genders.

Among the age categories (Table 3), the ≤44-years-old group had significantly lower scores than the ≥45-years-old group for vitality, role limitations due to emotional problems, mental health and Mental Component Summary (*p* < 0.05); on the physical function scale and the Physical Component Summary measurement, there were significant differences between age groups (*p* < 0.05), where older teachers had lower scores than the younger teachers.

### 3.3. Multivariate Regression Analysis

Table 4 presents the results of the logistical regression between the percentiles ≤50 (worse health) of the mental and physical health components as dependent variables, with sociodemographic variables as independent variables. There were two predictor conditions of a deterioration in the mental health component: first, teachers who indicated working more hours than before the pandemic (OR: 1.902; *p* < 0.05) and, second, those whose work–family balance was affected due to work demands (OR = 3.996; *p* < 0.05). Teachers in the first age group (≤44 years) presented greater risk (OR: 2.462; *p* < 0.01) of mental health deterioration than the second group (≥45 years). However, the younger teachers presented lower risk of deterioration in the physical health component (OR: 0.536; *p* < 0.05) than the older group.

## 4. Discussion

The objective of this study was to evaluate QoL in a sample of Chilean teachers and its association with their sociodemographic characteristics, work hours and work–family balance during the COVID-19 pandemic. The principal results of this study indicate that teachers present low scores in the mental QoL components during the COVID-19 pandemic. First, teachers ≤ 44 years old present a greater risk of effects on the mental QoL component, while teachers ≥ 45 years old present a greater risk of effects on the physical QoL component. Finally, teachers who perceived a negative impact on work–family balance, and teachers who indicated working more hours than before the pandemic, reported the greatest mental health impact risk.

### 4.1. Age and Quality of Life

Regarding age groups, in this study younger adult teachers (≤44 years) have a significant risk of mental health deterioration (OR: 2.462; *p* < 0.01), which coincides with the cases of teachers in China and Chile prior to COVID-19 [33,38,39] and during the COVID-19 pandemic [38,45]. These findings could be explained due to the reported fact that older teachers present lower stress because they are more competent in routine work and solve problems more independently [39]; however, in this study there is a lower physical deterioration risk in younger teachers (OR:0.536), a result which can be related to the high rate of physical conditions and significant work capacity reduction of teachers ≥45 years old compared to ≤44 years old reported in Bulgaria and Chile [33,46].

The results regarding mental health in younger teachers during the pandemic also align with working populations beyond teachers: for example, in the UK and Austria, there have been reports among young adult populations of major QoL impact and especially on mental health during COVID-19 [47,48], as well as among young health workers and young people in the general population in China [49,50], all associated with various health and social situations resulting from the progress of the pandemic [51]. These data indicate a strong mental health deterioration among younger teachers, which could be related to workload increasing during the pandemic. It could also indicate lower stress management ability in unfavorable conditions compared to older professionals. Although more experienced teachers have already been reported to have tools for better facing stress [39], this could also indicate the need to develop skills during teachers’ academic formation to face stress events related to their work.

### 4.2. Increase in Workload and Quality of Life

Various working groups, including teachers, have reported the need to reinvent their work routine to accommodate teleworking [6,17,21,22]. A total of 78.7% of teachers in this study indicated that they worked more hours during the pandemic than before it with the same contract, consequently presenting an increased risk of lower mental health quality (OR: 1.902). These results are even more relevant in relation to reports from the International Labour Organization [7], indicating that teleworking in the COVID-19 context contributes to a significant rise in mental health disorders, with risk factors including high workloads and rhythms, long working days, excessive task fragmentation and the perception of having to be available at all times, among other factors [7]. The findings of this study also agree with reports from Chilean teachers about mental health deterioration caused by working from home [52]. A study on Chilean teachers also found that teachers presented work burnout at a significantly greater level than other professions, with diminished engagement over the course of the nationwide health emergency due to the forced teleworking transition [27]. All this background is very important when we consider that pre-pandemic teachers already presented high rates of work overload, burnout and high rates of professional ailments [33,39,40,53,54].

### 4.3. Work Family Balance and Quality of Life

Regarding work–family balance in the teleworking context, our results show that 86% of teachers with low mental health scores indicated that their family and personal lives were more affected during the COVID-19 pandemic than before it began. This factor causes the greatest risk increase in presenting low mental health in this study (OR: 3.996; *p* < 0.001), results that coincide with reports about the impact telework has on the work–family balance from teachers, especially in female workers [55,56]. However, the findings of this study indicate that QoL was affected in both genders in its physical and mental summary components. Nevertheless, a significant decrease was observed in several dimensions of QoL: physical function, bodily pain, vitality and mental health (see Table 3). These data are consistent with recent studies where the QoL of female teachers during the global health crisis of COVID-19 was affected [38], where higher stress and anxiety have been reported compared to men [45]. These observations are relevant in the context that women are predominant in the teaching profession.

Furthermore, these findings can be compared with reports from other professions where an impact on the work–family balance is associated with a negative impact on workers’ mental health [57,58]. In the UK, it was reported that work overload and imbalance between work and personal lives are principal stressors [59]. Studies in Spain and China reported that work–family balance is an important source of stress and burnout [60,61]. Work overload during COVID-19 confinement could affect the work–family balance in this study, as demonstrated by reports from a Chinese national general population survey or in Japanese nursing schools [62,63]. Therefore, since work–family balance impact and work overload are stressors, they affect workers’ QoL, especially their mental health [56,57].

Finally, before the COVID-19 pandemic there were already reports of high work overload, stress, and lack of time for personal and family life with impact on teachers’ mental health [33,38,54,64]. This means that all extra work in teaching hours, e.g., material preparation, test creation and revision and administrative work [53], are intensified during Chilean teachers’ teleworking, with a high risk of mental health deterioration in their QoL.

### 4.4. Limitations

The limitations of this study are typical of a cross-sectional study without the ability to compare results before and during the pandemic. Measuring impacts on the new work context on family and work life was also done via questions, making it necessary to have more in-depth studies which can evaluate the factors affecting work–family balance in teachers. Besides, the study is limited by using a single yes/no question to examine whether work/family relationships were affected during the COVID-19 pandemic. In addition, the variable number of school-age children of teachers was not included and it is a variable that deserves future studies due to its reported influence on teachers’ mental health [45]. Another limitation is the snowball sampling method; non-probability sampling has inherent limitations in representing the entire population. However, it has the advantage of covering many people. The snowball sampling also had an important reach, but most representation came from the central zone of Chile, which may be because most of the national population lives in this zone. Future studies should have larger samples more representative of the regions of Chile.

## 5. Conclusions

The COVID-19 pandemic and the unavoidable change in Chilean teachers’ work format intensified work overload, generated work–family balance conflicts and resulted in a negative effect on teachers’ mental QoL components. Teachers ≤ 44 years old had the highest risk in the mental health component and teachers ≥ 45 years old had the highest physical health component risk. This suggests promoting improvements in policies for distributing teachers’ contractual hours, considering all the extra work hours which are unpaid but essential for making and reviewing material [53], in order to reduce pressure, work overload and burnout, factors which increase the risks associated with QoL deterioration. In addition, we agree with recent research that recommends establishing public policies focused on mitigating these stressors beyond the human-information and communication technologies interaction [65]. We also suggest that undergraduate teachers be adequately trained to face the conflicts associated with the profession.

## Figures and Tables

**Table 1:** Sociodemographic and working characteristics of Chilean teachers separated by gender and age group.

	Total Sample	Gender		*p*-Value	Age		*p*-Value
		Male	Female		≤44	≥45	
	*n* (%)	*n* (%)	*n* (%)		*n* (%)	*n* (%)	
Age (years) ^c^	37.57 ± 10.75	38.39 ± 11.21	37.33 ± 10.63	0.442 ^a^	32.27 ± 5.48	53.67 ± 5.52	<0.001 ^a^
Marital status							
Single	158 (47.02)	31 (43.66)	127 (47.92)	0.812 ^b^	140 (55.34)	18 (21.69)	<0.001
Married/partnered	155 (46.13)	35 (49.30)	120 (45.28)		108 (42.69)	47 (56.63)	
DWW	23 (6.85)	5 (7.04)	18 (6.79)		5 (1.98)	18 (21.69)	
Experience work (years) ^c^	23.62 ± 14.41	27.01 ± 14.77	22.71 ± 14.21	0.278 ^a^	26.72 ± 9.34	22.60 ± 15.61	<0.01 ^a^
Type of contract							
Fixed-term	218 (64.88)	22 (30.99)	96 (36.23)	0.411 ^b^	122 (44.27)	6 (7.23)	<0.001
Indefinite-term	118 (35.12)	49 (69.01)	169 (63.77)		141 (55.73)	77 (92.77)	
Type of school							
Public (state)	100 (29.76)	21 (29.58)	79 (29.81)	0.589 ^b^	74 (29.25)	26 (31.33)	0.813 ^b^
Private (subsidized)	156 (46.43)	30 (42.25)	126 (47.55)		120 (47.43)	36 (43.37)	
Private (non-subsidized)	80 (23.81)	20 (28.17)	60 (22.64)		59 (23.32)	21 (25.30)	
Domestic work							
<15 h	268 (79.76)	60 (84.51)	208 (78.49)	0.262 ^b^	208 (82.21)	60 (72.29)	0.059 ^b^
>15 h	68 (20.24)	11 (15.49)	57 (21.51)		45 (17.79)	23 (27.71)	
Work hours in pandemic ^d^							
Higher	258 (78.66)	51 (72.86)	207 (80.23)	0.182 ^b^	191 (76.40)	67 (85.90)	0.126 ^c^
Less/Equal	70 (21.34)	19 (21.14)	51 (19.77)		59 (23.6)	11 (14.1)	
Work–family balance							
Unaffected	46 (14.02)	11 (15.71)	35 (13.57)	0.646 ^b^	32 (12.80)	14 (17.95)	0.253 ^b^
Affected	282 (85.98)	59 (84.29)	223 (86.43)		218 (87.20)	64 (82.05)	

DWW, Divorced Widow Widower; <15 and >15, domestic work in hours; *p* < 0.05. ^a^ Wilcoxon test, ^b^ Chi-squared; ^c^ Data are expressed as mean ± standard deviation; ^d^ Work hours in pandemic, Higher: more than before the pandemic, Less/Equal: less than or equal before the pandemic.

**Table 2:** Sociodemographic and working characteristics of Chilean teachers separated by PCS (Physical Component Summary) and MCS (Mental Component Summary) scores below 50th percentile and above 50th percentile.

	Physical Component Summary		*p*-Value	Mental Component Summary		*p*-Value
	<P50	>50		<P50	>P50	
	*n* (%)	*n* (%)		*n* (%)	*n* (%)	
Age (years) ^c^	38.95 ± 0.89	36.17 ± 0.79	0.022 ^a^	34.60 ± 0.71	40.51 ± 0.88	<0.001 ^a^
≤44	116 (69.05)	137 (81.55)	0.010 ^b^	141 (83.93)	112 (66.7)	<0.001 ^b^
≥45	52 (30.95)	31 (18.45)		27 (16.07)	56 (33.33)	
Marital status						
Single	75 (44.64)	83 (49.40)	0.140 ^b^	89 (52.98)	69 (41.07)	0.091 ^b^
Married/partnered	77 (45.83)	78 (46.43)		69 (41.07)	86 (51.19)	
DWW	16 (9.52)	7 (4.17)		10 (5.95)	13 (7.74)	
Work experience (years) ^c^	24.11 ± 13.0	23.13 ± 14.85	0.571 ^a^	24.58 ± 15.32	22.65 ± 13.42	<0.001 ^a^
Type of contract						
Fixed-term	62 (36.91)	56 (33.33)	0.493 ^b^	77 (45.83)	41 (24.40)	<0.001 ^b^
Indefinite-term	106 (63.10)	112 (66.67)		91 (54.17)	127 (75.60)	
Type of school						
Public (state)	55 (32.74)	45 (26.79)	0.489 ^b^	55 (32.74)	45 (26.79)	0.109 ^b^
Private (subsidized)	75 (44.64)	81 (48.21)		81 (48.21)	75 (44.64)	
Private (non-subsidized)	38 (22.62)	42 (25)		32 (19.05)	48 (28.57)	
Domestic work						
<15 h	135 (80.36)	133 (79.17)	0.786 ^b^	138 (82.14)	130 (77.38)	0.342 ^b^
>15 h	33 (19.64)	35 (20.83)		30 (17.86)	38 (22.62)	
Work hours in pandemic ^d^						
Higher	133 (82.10)	125 (75.30)	0.133 ^b^	141 (84.33)	117 (72.67)	0.010 ^b^
Less/Equal	29 (17.9)	41 (24.70)		44 (27.33)	44 (27.33)	
Work–family balance						
Unaffected	17 (10.49)	29 (17.47)	0.069 ^b^	10 (5.99)	36 (22.36)	0.001 ^b^
Affected	145 (89.51)	137 (82.53)		157 (94.01)	125 (77.64)	

DWW, Divorced Widow Widower; *p* < 0.05. ^a^ Wilcoxon test, ^b^ Chi-squared; ^c^ data are expressed as mean ± standard deviation. ^d^ Work hours in pandemic, Higher: more than before the pandemic, Less/Equal: less than or equal before the pandemic.

**Table 3:** Comparisons between median values of eight scales and mental and physical QoL components by SF-36 according to gender and age group.

QoL	Total Sample	Gender		*p*	Age		*p*
	Mean ± SD	Male	Female		≤44	≥45	
PF	49.42 ± 7.09	51.14 ± 6.68	48.96 ± 7.14	0.004 ^a^	50.56 ± 6.20	45.96 ± 8.44	<0.001 ^a^
RP	45.46 ± 7.08	44.91 ± 6.92	45.60 ± 7.12	0.476 ^a^	45.53 ± 7.23	45.25 ± 6.62	0.835 ^a^
BP	39.94 ± 9.78	43.73 ± 8.98	38.92 ± 9.75	<0.001 ^a^	39.71 ± 9.95	40.63 ± 9.24	0.607 ^a^
GH	43.44 ± 10.04	43.91 ± 9.00	43.31 ± 10.31	0.802 ^a^	43.46 ± 10.04	43.35 ± 10.09	0.897 ^a^
VT	39.80 ± 8.39	43.40 ± 8.39	38.83 ± 8.13	<0.001 ^a^	39.02 ± 8.01	42.14 ± 9.11	<0.001 ^a^
SF	35.60 ± 11.83	35.70 ± 11.90	35.58 ± 11.84	0.976 ^a^	35.89 ± 11.22	34.72 ± 13.57	0.442 ^a^
RE	43.76 ± 7.28	44.11 ± 7.20	43.67 ± 7.32	0.656 ^a^	43.01 ± 7.15	46.06 ± 7.25	<0.001 ^a^
MH	35.31 ± 10.02	38.51 ± 10.84	34.45 ± 9.63	0.008 ^a^	34.69 ± 9.31	37.18 ± 11.80	0.045 ^a^
PCS	47.58 ± 6.84	48.79 ± 6.74	47.26 ± 6.85	0.119 ^a^	48.28 ± 2.77	45.46 ± 6.67	0.002 ^a^
MCS	35.66 ± 9.48	37.54 ± 10.07	35.15 ± 9.27	0.059 ^b^	34.68 ± 9.03	38.63 ± 10.24	<0.001 ^a^

SD, standard deviation; QoL, quality of life; PF, physical function; RP, role limitations due to physical problems. RE, role limitations due to emotional problems; BP, bodily pain; SF, social functioning; VT, vitality; GH, general health perceptions; MH, mental health; PCS, Physical Component Summary; MCS, Mental Component Summary; *p* < 0.05 ^a^ Wilcoxon’s text, ^b^ *t*-test; *p* < 0.05.

**Table 4:** Logistical regressions for association of the 50th percentile of the Physical Component Summary and Mental Component Summary with sociodemographic and work characteristics adjusted by gender and age.

	Physical Component Summary			Mental Component Summary		
	Percentile 50			Percentile 50		
	Model 1	Model 2	Model 3	Model 1	Model 2	Model 3
	OR	OR	OR	OR	OR	OR
	*p*-Value	*p*-Value	*p*-Value	*p*-Value	*p*-Value	*p*-Value
Work in pandemic (more than before)	1.398	1.390	1.268	1.747	1.733	1.902
	0.227	0.236	0.401	0.052	0.056	0.029
Work–family Relation (affected)	1.690	1.719	1.866	4.119	4.252	3.996
	0.115	0.105	0.067	<0.001	<0.001	<0.001
Domestic work (>15 h)		0.827	0.761		0.724	0.778
		0.501	0.340		0.261	0.393
Gender (female)			1.397			1.350
			0.228			0.295
Age (≤44 years)			0.536			2.462
			0.021			0.001
Hosmer–Lemeshow test ^a^	0.891	0.999	0.177	0.443	0.580	0.320

^a^ A value above 0.05 indicates that the model fits the data.

## Data Availability

The datasets generated and/or analyzed during the current study are available from the corresponding author on reasonable request.
